# A novel approach to the clustering of microarray data via nonparametric density estimation

**DOI:** 10.1186/1471-2105-12-49

**Published:** 2011-02-08

**Authors:** Riccardo De Bin, Davide Risso

**Affiliations:** 1 Department of Statistical Sciences, University of Padova, Padova, Italy

## Abstract

**Background:**

Cluster analysis is a crucial tool in several biological and medical studies dealing with microarray data. Such studies pose challenging statistical problems due to dimensionality issues, since the number of variables can be much higher than the number of observations.

**Results:**

Here, we present a general framework to deal with the clustering of microarray data, based on a three-step procedure: (i) gene filtering; (ii) dimensionality reduction; (iii) clustering of observations in the reduced space. Via a nonparametric model-based clustering approach we obtain promising results both in simulated and real data.

**Conclusions:**

The proposed algorithm is a simple and effective tool for the clustering of microarray data, in an unsupervised setting.

## Background

The analysis of gene expression microarray data using clustering techniques plays an important role, for instance, in the discovery, validation, and understanding of various classes and subclasses of cancer [1]. There are two ways of clustering a gene expression matrix [2,3]: (i) gene function may be inferred from clusters of genes similarly expressed across the samples and (ii) samples can form groups which show similar expression across the genes. Moreover, genes and samples can be clustered simultaneously, with their inter-relationship represented by bi-clusters [4,5].

The clustering of the genes on the basis of the samples is a standard cluster analysis problem that can be effected by a variety of algorithms [1]. For a comprehensive review see [2].

A more challenging problem is the clustering of the samples on the basis of the genes, where the standard clustering techniques, such as *k*-means or hierarchical clustering, fail to capture complex local structures, due to the high-dimenionality of the data [2].

In recent years, computational improvement enabled new clustering techniques and contributed to the development of previously unfeasible methods. In this context, McLachlan et al. [1] propose a mixture model-based approach to cluster microarray expression data. Their scheme accounts for gene selection through mixtures of *t *distributions, and dimensionality reduction through a mixture of factor analyzers. More precisely, they select a gene on the basis of a likelihood ratio statistic for testing one versus two components in the mixture model. In the second step of their algorithm, they cluster the samples by fitting a two-component mixture of factor analyzers.

Although their method sounds like a good approach to clustering samples in a high-dimensional space, there are three main limitations. Firstly, the parametric assumptions about clusters distributions can be restrictive [6]; for example, two Gaussian random variables can result in a single mode (one cluster) or even a two component multivariate Gaussian mixture can lead to more than two modes [6]. Moreover, it requires pre-specification of the number of the mixture components; this represents a serious limitation from an unsupervised perspective, which assumes that the true number of clusters is unknown. Finally, the number of parameters per component grows as the square of the dimension of the data [7], this is a major shortcoming in high-dimensional data.

In this paper, we present a novel strategy, which consists in applying a clustering technique after gene filtering and dimensionality reduction, in order to exploit the most significant dimensions in the definition of the clusters. Our procedure can be thought of as a three-step algorithm: (i) gene filtering; (ii) dimensionality reduction; (iii) clustering in the reduced space.

Several authors outlined the importance of a gene filtering step prior to inferential procedures [8] or cluster analysis [9]. Tritchler et al. [9] empirically showed that principal components and cluster analysis are strongly affected by gene selection, and that filtering out uninformative genes can reduce bias in the clustering of samples. Furthermore, Johnstone and Lu [10] showed, from a theoretical point of view, that some initial reduction in dimensionality is desirable before applying a principal component analysis, when *p *is larger than *n*.

Traditional approaches to gene filtering are based on thresholding the mean or the variance of genes across samples. Bourgon et al. [8] found that gene-by-gene filtering by overall variance increased the power of the subsequent *t*-test. Tritchler et al. [9] considered the covariance structure of the genes, defining filters that preserve the topology of the network.

Nevertheless, from a clustering point of view, these approaches could be unsafe: a gene should be considered relevant if it is important in the definition of the clusters; therefore, it seems more appropriate to retain those genes whose univariate distribution highlights a clear grouping among the observations rather than the ones with higher variance.

To evaluate our general strategy, we implement an algorithm based on a nonparametric model-based clustering technique by Azzalini and Torelli [11], which we will refer to as *pdfCluster *(see the Methods Section for a brief introduction). We compare it with a traditional partition algorithm (i.e., *k-means*), and with a similar strategy in which we use, instead of *pdfCluster*, its direct competitor, *Mclust *[7,12], a state-of-the-art mixture-model-based clustering tool. By using the nonparametric approach based on *pdfCluster*, we achieve improvements in clustering of samples both in simulated and in real experiments. To be consistent with microarray applications, we use here the typical microarray terminology: we denote by "genes" the *p *variables and by "samples" the *n *observations. Nonetheless, it should be clear that the proposed approach is not limited to microarray data, but, in principle, it could be applied to every set of continuous variables with "large *p*, small *n*".

## Results and Discussion

### A novel algorithm to clustering of expression data

As we said, clustering samples using expression data is a challenging statistical problem due to dimensionality issues. Therefore, in this context, it is unfeasible to directly apply a clustering technique to the whole data matrix. Here, we evaluate our strategy implementing an algorithm which exploits the self-detection of number of clusters feature of *pdfCluster*.

The algorithm can be summarized as follows: (i) cluster samples using the univariate distribution of each gene and select for the subsequent analyses the *p' *genes, in which *pdfCluster *identifies two or more clusters; (ii) reduce dimensionality by selecting the first *p" *principal components; (iii) apply *pdfCluster *in the *p"*-dimensional space. It is straightforward to see that this algorithm falls within the general framework defined in the Background Section.

**Figure 1:**
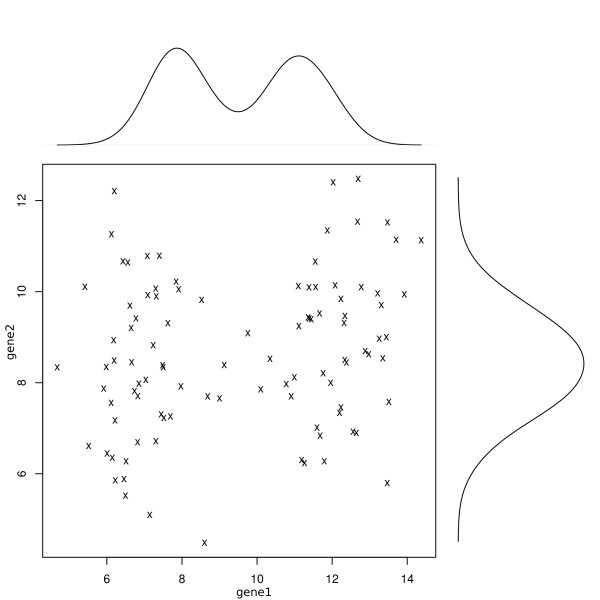
**Example of gene selection in the filtering step of our procedure**. Gene1 is crucial in cluster definition, while gene2 is not. The univariate distributions of the genes reflect this, as one can see from the Gaussian kernel density estimation reported along the axes.

As for step (i), i.e., *gene filtering*, we consider a gene relevant if its values in one category (healthy, say) are different from the ones in the other category (unhealthy, say) or categories. From another point of view, this means that the samples representing the healthy subjects are separated from the unhealthy ones, or, more simply, the samples are in different clusters. In this way, it seems reasonable to apply a cluster method to each gene, and retain as relevant those genes for which the method identifies distinct clusters. In a nonparametric framework, we can apply *pdfCluster *to each gene, taking advantage of the self-detection number of clusters feature. We select only the genes for which the method detects two or more clusters (see Figure 1). We consider a clustering technique to define informative genes over the traditional variance-based approaches, because small overall variance does not necessarily imply a single cluster, and high overall variance is not always an indication of two of more clusters. Moreover, variance-based filters depend on the choice of an arbitrary threshold, which can be difficult to choose, since one typically does not know which portion of the genes is responsible for the clustering of the samples. As for step (ii), i.e., *dimensionality reduction*, considered if the selected genes are still too many, we propose to keep the first principal components, as in [11]. The principal component analysis is a very simple procedure which reduces the dimension of a data set of a large number of interrelated variables, preserving as much as possible of the data set variation. Since it has no requirements about the data distribution, it is consistent with our nonparametric strategy.

In order to compare our approach to *Mclust*, we carried out a procedure analogous to the one described here, but using the normal-mixture model both in step (i) and (iii). Note that this procedure differs from the one in McLachlan et al. [1], because they use a mixture of factor analyzers to select genes and reduce dimensionality.

#### Computational issues

The further dimensionality reduction in our step (ii) is necessary since *pdfCluster*, in order to compute the Delaunay triangulation, exploits the *Quickhull *algorithm [13]. Barber et al. [13] state that the Quickhull algorithm for finding the convex hull of a set of *n *points in ℝ^*p *^requires at most *O*(*n *log *n*) operations if *p *≤ 3, and *O*(*n^m^/m*!) where *m *= ⌊*p*/2⌋ for *p *> 3. Azzalini and Torelli [11] observed that the computing time increases less than quadratically in *n *for any fixed *p*, but it increases more than exponentially in *p *for fixed *n*. Our experience is that for *p *= 1, the algorithm is very fast (less than one minute to run 20,000 times with an Intel(R) Core(TM)2 Quad CPU Q9400 @ 2.66 GHz). Therefore, there are no computationally related problems for step (i). Moreover, with *p *< 10 it takes a reasonable time (e.g. 11 mins in our machine with *p *= 9) to complete the procedure.

#### Number of principal components

In order to choose the number of principal components, we carried out a small simulation study (data not shown): we found that, with *n *= 100, *pdfCluster *performs at best, in terms of misclassification error, with 3-4 dimensions, while with *p *= 5 the misclassification error starts to grow. This is probably due to the extreme dispersion of the observations in higher dimensional spaces (the well-known curse of dimensionality). The performances of *pdfCluster *are slightly better with 4 components, but the improvement does not justify the increased computational time (recall that the order of the number of operations needed to compute the Quickhull algorithm massively changes between *p *≤ 3 and *p *> 3). Thus, for the subsequent analyses, we will retain 3 principal components.

### Simulated data

In this Section, we evaluate our proposal by means of simulated data. For simulating data with structure similar to that of real microarray experiments, we use two schemes, i.e., the Gamma-Gamma (GG) model [14] and the Normal-Uniform (NU) model [15].

In GG model we simulate data with two clusters (e.g. case/control), such that the majority of genes are equally expressed between the groups and a small fraction of them (5%) is differentially expressed. This mimics a classical experiment in which some diseased subjects are compared to healthy controls.

In NU model we simulate data with three clusters: *cluster 1 *consists of 40 samples with 150 up-regulated and 50 down-regulated genes; *cluster 2 *consists of 40 samples with 50 down-regulated genes; *cluster 3 *consists of 20 samples with neither up- nor down-regulated genes. Note that cluster 2 and 3 are "closer" to each other than to cluster 1 and that cluster 3 has smaller sample size. This mimics a more elaborate design, e.g. two different types of a specific disease versus a normal control.

#### GG model

Table 1 shows that both *pdfCluster *and *Mclust *provide results surprisingly accurate in correct cluster recognition, low error rate and high sensitivity/specificity: this could be explained by an extreme distance between the two groups in the original *p*-dimensional space.

**Table 1:** Simulation results for GG model

	*pdfCluster*		*Mclust*	
	**mean**	**se**	**mean**	**se**

SE	0.9877	0.0007	0.9991	0.0001
SP	0.9866	0.0008	0.9985	0.0004
ER	0.0128	0.0006	0.0012	0.0002
RG	0.0837	0.0061	0.7787	0.0093

CC	0.77		0.84	

Simulation results for *pdfCluster *and *Mclust *in GG model: rate of correct identification of number of clusters (CC), sensitivity (SE), specificity (SP) and error rate (ER) in the classification of the samples, and error rate in the selection of relevant genes (RG).

More interesting is the very different behaviour in the choice of the relevant genes: *pdfCluster *is very good in recognizing them, with a very low error rate (about 8%), while *Mclust *shows a very high error rate (about 78%). We simulated a relatively small number of "marker" genes; *pdfCluster *correctly discards the majority of genes as non-relevant in the determination of the clusters, while *Mclust *seems to be too sensitive to outliers, erroneously capturing differences due to random noise.

#### NU model

As expected, Table 2 shows that in this model both *pdfCluster *and *Mclust *lead to higher classification errors than in GG model. Also in the gene filtering step, both methods have difficulties in finding the relevant genes.

**Table 2:** Simulation results for NU model

	*pdfCluster*		*Mclust*	
	**mean**	**se**	**mean**	**se**

RG	0.433	0.041	0.616	0.077

CC2	0.47		0.34	

CC3	0.19		0.39	

ER	0.135	0.004	0.227	0.005

Simulation results for *pdfCluster *and *Mclust *in NU model: rate of two clusters identification (CC2), rate of three clusters identification (CC3), error rate in the classification of samples (ER) and error rate in the selection of relevant genes (RG).

*Mclust *is able to recognize three clusters in 39% and two clusters in 34% of the simulations; *pdfCluster *recognizes three clusters in 19% and two clusters in 47% of the simulations. On the other hand, the mean error rate of the final classification is 0.135 for *pdfCluster *while for *Mclust *is 0.227. This is probably due to the fact that the cases in which *pdfCluster *correctly recognizes three clusters are those with the most separated clusters among the ones recognized by *Mclust*: obviously, in the less separated clusters cases, it is more difficult to allocate the samples.

Finally, it is worth noting that *pdfCluster *outperforms *Mclust *according to the gene selection error rate ("RG" row): as in previous simulation study, *pdfCluster *works better in recognizing which genes are effectively responsible for the determination of the clusters.

#### Sample Size

One issue with microarray data is often the low sample size. In order to evaluate its effect on the performance of our approach, we simulated data from the NU model, varying the sample size *n*. For different values of *n*, namely *n *= 10, 20, 50, 100, 200, we simulated *B *= 1,000 samples in a setting similar to the previous Section, i.e., 40% of the observations forming cluster 1, 40% forming cluster 2 and 20% cluster 3.

Table 3 shows the misclassification error rate for both *pdfCluster *and *Mclust. Mclust *performs badly for low and moderate sample size (*n *≤ 50), reaching results comparable to that of *pdfCluster *only with a high number of observations (*n *= 200), which is rare in microarray studies. On the other hand, *pdfCluster *behavior is stable across different sample sizes, yielding good results even when *n *≤ 20.

**Table 3:** Sample size

ER	*pdfCluster*		*Mclust*	
**n**	**mean**	**se**	**mean**	**se**

10	0.182	0.033	0.302	0.039
20	0.131	0.030	0.381	0.025
50	0.114	0.020	0.287	0.025
100	0.137	0.015	0.230	0.019
200	0.172	0.012	0.204	0.014

Misclassification error rate (ER) for *pdfCluster *and *Mclust *in NU model, varying the sample size.

### Real data

Along with simulations, we consider two benchmarking real datasets, studied before by several authors [1,16-20], which we will refer to as the Colon data and the Leukaemia data (see Method Section for details on the datasets).

#### Colon data

As described above, we analyze the dataset, following three steps: (i) gene filtering, (ii) dimensionality reduction, (iii) clustering of samples. Namely, the first step of the procedure consists in applying the cluster algorithm to the univariate distribution of each gene. The genes that show two or more clusters are considered for the further steps.

In the first step, the *pdfCluster *algorithm is able to recognize 84 genes, which discriminate data into two or more groups. We proceed by considering the first three principal components of this reduced data-matrix. The procedure finds three clusters, summarized in Table 4 which clearly correspond to biologically meaningful groups. The first cluster consists of tumor tissues (with 3 misclassified samples), while clusters 2 and 3 comprise normal tissues (with 5 misclassified). It is worth noting that six out of the eight misallocated samples (tumor tissues 30, 33 and 36 and normal tissues 48, 58 and 60) are found to be misclassified in several previous analyses, including [1,17]. As stated, for instance, in [17], these six samples are likely to be wrongly labeled. Furthermore, Getz et al. [19] reported that there was a change in the protocol during the experiments: tumor samples 1-11 and normal samples 41-51 were collected within the first protocol, while tumor samples 12-40 and normal samples 52-62 were collected within the second. Although for the tumor samples our approach did not recognize any difference between the protocols, cluster 2 and cluster 3 split normal tissues in two groups according to the protocols.

**Table 4:** Clusters found in Colon data

Cluster 1	1-6,8-19,21-29,31,32,34,35,37-40,48*,58*,60*
Cluster 2	**7**,41-47,49-51,52
Cluster 3	20,30*,33*,36*,53-57,59,61,62

Clusters found after *pdfCluster *procedure in Colon data; tumor samples are labeled 1-40, normal samples 41-62; misallocated samples are shown in bold. The star represents a wrongly labeled samples.

In the first step, *Mclust *is able to find 369 discriminant genes. We consider the first three principal components of this sub-space for clustering. The procedure finds two clusters, with a rather high misclassification error (see Table 5). We also apply the *k-means *algorithm to the entire dataset. The results of the three approaches are shown in Table 5. It can be seen that *k-means*, exploited in the original *p*-dimensional space, does not perform well. Moreover, *pdfCluster *outperforms (in terms of error rate) *Mclust*, if one considers cluster 2 and 3 together as the normal samples.

**Table 5:** Confusion matrices for Colon data

	*pdfCluster*		*Mclust*		*k-means*	
Real	1	2-3	1	2	1	2
Tumor	35	5	29	11	23	17
Normal	3	19	12	10	6	16
ER:	0.13		0.37		0.37	

Confusion matrices for *pdfCluster, Mclust *and *k-means *with error rates (ER) for Colon data.

As stated before, McLachlan et al. [1] studied the same microarray dataset. They selected 446 relevant genes, achieving clusters that seem to recognize the change of protocol in the data structure, but fail to recognize the normal/tumor differences [1]. Nevertheless, they achieved results slightly better than ours (ER = 0.1) considering a particular subspace: they clustered genes in 20 groups and considered only the second group (consisting of 24 genes) to cluster data [1]. Although this approach leads to good results in this example, it seems difficult to reproduce the procedure in an unsupervised setting.

#### Leukaemia data

As stated in [18], the Leukaemia dataset presents two different problems: an easier one, consisting of separating ALL from AML (two-class problem, hereafter) and a harder one, consisting also of recognizing the differences in B-cell and T-cell subclasses (three-class problem).

Again, we consider the strategy previously described. In the filtering step, *pdfCluster *recognizes 313 discriminant genes. Note that the higher number of genes selected with respect to Colon data is consistent with the higher difficulty of the problem. We proceed by considering the first three principal components of this subspace. The *pdfCluster *algorithm finds two clusters, which clearly represent ALL and AML samples, with 4 AML samples classified as ALL and 5 ALL samples classified as AML, leading to a misclassification error rate of 0.125 (Table 6): *pdfCluster *is able to solve the two-class problem, but it misses the three-class problem.

**Table 6:** Confusion matrices for Leukaemia data

	*pdfCluster*		*Mclust*				*k-means*		
Real	1	2	1	2	3	4	1	2	3
ALL B-cell	37	1	9	20	9	0	15	0	23
ALL T-cell	5	4	0	0	7	2	7	2	0
AML	4	21	0	2	1	22	1	23	1

Confusion matrices for *pdfCluster, Mclust *and *k-means *with error rates (ER) for Leukaemia data.

In the first step, *Mclust *fails to select relevant genes, recognizing 3,119 out of 3,892 genes as discriminant among the groups. Based on the first three principal components of the subspace spanned by these genes, *Mclust *clusters samples in four groups. We could interpret the merged clusters 1-2 as the ALL B-cell class, and cluster 4 as the AML class, while cluster 3 interpretation is less clear (Table 6). Although *Mclust *is able to find more than two clusters, it fails to distinguish between B-cell and T-cell classes, leading to hardly interpretable clusters.

The Leukaemia dataset has been studied by McLachlan et al. [1] as well. The authors found 2,015 relevant genes after the variable selection step. For the two-class problem, their results were very good (only one sample misallocated), but they failed to solve the three-class problem.

It should be noted that, unlike our algorithm, the procedure used in [1] needs prior specification of the number of clusters, which is not desirable in an unsupervised learning, especially in cancer tissue classification, where one of the main goals is to find new subclasses of tumors.

## Conclusions

Model-based approaches to clustering of data have received increasing attention in the last few years, as they provide a sound mathematical-based method. Unfortunately, in microarray applications, the high dimensionality of the space makes the clustering of samples in the whole space unfeasible within a model-based framework.

Here, we have discussed a general strategy for the clustering of microarray expression data, based on gene filtering and dimensionality reduction as preliminary steps in the cluster analysis.

We have discussed a nonparametric density estimation-based algorithm within this framework, showing promising results both in simulated data and in two real applications, with surprisingly good computational performances.

In our simulation experiments, we have found that *pdfCluster *leads to slightly better performances than *Mclust*. Moreover, the gene filtering step is much more effective using *pdfCluster *than using *Mclust *both in simulated and in real datasets. Here, "effective" means good results in terms of both dimension reduction (e.g. in Leukaemia data *pdfCluster *selected 313 genes versus the 3,119 selected by *Mclust*) and of correct selection (e.g. in GG model the gene selection error rates are 0.08 and 0.77, respectively).

Here we have used *pdfCluster *in order to select relevant genes. We underlined the assets of this chioce, but it is clear that any unsupervised technique able to discard the irrelevant genes can be used. Similarly, the choice of principal component analysis in the dimensionality reduction step is only one among several possible choices. Since there are no guarantees that the first principal components preserve the cluster structure in the reduction of original dimension of data [21], future efforts could be made in trying different approaches, such as the projection pursuit [21,22] or the principal curves [23]. Nevertheless, in our case the principal component analysis gives good results and provides a low dimensional dataset on which it is feasible to apply a model-based technique such as *pdfCluster*.

All the statistical analyses and simulations have been performed with R [24] and with a public domain implementation of the "Quickhull" algorithm [13] available at http://www.qhull.org/.

The datasets used are both freely available as Bioconductor [25] packages ("colonCA" for Colon data and "golubEsets" for Leukaemia data).

## Methods

### Simulation models

#### GG model

The samples are assumed to be independently generated from Gamma distributions with a constant shape parameter *α *and gene-specific random scale *λ_i_*, *i *= 1, ..., *p*; *λ_i _*is assumed to have a Gamma distribution with shape hyperparameter *α*_0 _and scale hyperparameter *ν*. The genes are generated to be either "equally expressed" (i.e. one group) or "differentially expressed" (i.e. two groups) among the samples. We generated *n *= 100 samples and *p *= 2,000 genes, each with probability 0.05 of being differentially expressed. We fixed parameter values as suggested by [26]. We applied our algorithm to the data matrix obtained, selecting a number of relevant genes and using the first three principal components as input for the *pdfCluster *algorithm. We repeated this procedure *B *= 5,000 times.

#### NU model

The model deals with *k*-class classification of samples, for general *k*. It is based on a mixture of Normal and Uniform distributions. We exploit the model to simulate gene expressions for a three-class problem, similar to that of the leukaemia data.

Let us denote with *x_ji _*the measured intensity of gene *j *in sample *i*, *j *= 1, ..., *p*, *i *= 1, ..., *n*. We define three categories from which *x_ji _*can arise and use *e_ji _*to represent them. (i) *e_ji _*= -1, i.e., gene *j *has abnormally low expression in sample *i *(down-regulation); (ii) *e_ji _*= 0, i.e., gene *j *has normal expression in sample *i*; (iii) *e_ji _*= 1, i.e., gene *j *has abnormally high expression in sample *i *(up-regulation). For each gene *j*,

$$x_{ji}|(e_{ji}=e)\sim f_{e,j},\;e\in \left\{-1,0,1\right\}.$$

Following [15], we use a Uniform distribution for *f*_-1,*j *_and *f*_1, *j *_and a Normal distribution for *f*_0,*j*_. More specifically,

$$\begin{array}{c} f_{-1,j}=U(-\kappa_{j}+\alpha_{i}+\mu_{j},\alpha_{i}+\mu_{j}),\\ f_{0,j}=N(\alpha_{i}+\mu_{j},\sigma_{j}),\\ f_{1,j}=U(\alpha_{i}+\mu_{j},\alpha_{i}+\mu_{j}+\kappa_{j}), \end{array}$$

where *μ_j _*represents the gene-effect and *α_i _*the sample-effect for the normal expression level (see [15] for details). The authors justify the choice of the distributions arguing that, for normally expressed genes, the differences in observed values are due mainly to noise introduced in the experimental stage, while the Uniform distribution may reflect the failure of a biological mechanism that controls the expression level.

We simulated data from the model in a hierarchical framework, with the following initial parameter values:

$$\begin{array}{c} \mu_{j}\sim N(7.5,1.5),\\ \sigma_{j}^{-1}\sim G(2,1),\\ \alpha_{i}\sim N(0,1),\\ \kappa_{j}\sim ℰ(1)+7\sigma_{j}, \end{array}$$

where G denotes the Gamma and ℰ the Exponential distribution. We simulated *B *= 5,000 datasets of *n *= 100 samples, *p *= 1,000 genes and *m *= 3 clusters defined as follows: *cluster 1 *consists of 40 samples with 150 up-regulated and 50 down-regulated genes; *cluster 2 *consists of 40 samples with 50 down-regulated genes; *cluster 3 *consists of 20 samples with neither up- nor down-regulated genes.

### Real data

#### Colon data

Alon et al. [16] used Affymetrix oligonucleotide arrays to measure the expression of 6,500 human genes in 40 tumor and 22 normal colon tissue samples. They focused on the subset of 2,000 genes with highest minimal intensity across the samples: the raw expression values of these 2,000 genes comprise our dataset. Following notation in [1], we named 1-40 the tumor samples and 41-62 the normal samples. Before clustering the tissues, we pre-processed the raw intensities taking the logarithm and applying the quantile normalization [27], which is a standard choice for single-channel microarray technology.

#### Leukaemia data

Golub et al. [20] studied the gene expression of two types of acute leukaemias, acute lymphoblastic leukaemia (ALL) and acute myeloid leukaemia (AML). Gene expression levels were measured using Affymetrix oligonucleotide arrays containing 6,817 human genes. The dataset comprises 47 cases of ALL (38 B-cell and 9 T-cell) and 25 cases of AML. The classification of samples is more difficult in this example than in Colon data because it is much harder to classify between subclasses of the same plasticity than to distinguish between healthy and cancer tissues. Moreover, we have a typical hierarchical structure, since B-cell and T-cell are subclasses of the ALL class and are harder to separate than AML and ALL. Following [18], three preprocessing steps are applied to the intensity matrix: (a) thresholding, floor of 100 and ceiling of 16,000; (b) filtering, exclusion of genes with max/min ≤ 5 or (max - min) ≤ 500; (c) base 10 log transformation. This procedure left us with 3,892 genes.

### Evaluation criteria

Both in simulated and in real data, we evaluate the performances of the methods by calculating the error rate (proportion of misclassified samples, ER), the sensitivity (SE) and the specificity (SP). Moreover, in the simulation studies, we record the frequency with which each method finds the correct number of clusters (CC), and we evaluate the performance of the methods in selecting discriminant genes, considering the error rate in the classification of relevant genes (RG), knowing *a priori *which genes have been generated to have different values among the groups.

Since cluster 2 and 3 of the Normal-Uniform model have been simulated to be close to each other, in this model we also consider the number of times in which each method is able to recognize two clusters (cluster 1 versus clusters 2-3) or three clusters.

### The pdfCluster algorithm: an overview

In the literature, nonparametric cluster analyses based on mode identification have already been presented. See [6,7,28-30]. The *pdfCluster *algorithm [11] starts from a quite simple idea, introduced by Hartigan in 1975 [31], who stated:

Clusters may be thought of as regions of high density separated from other such regions by regions of low density.

These regions are achieved by "cutting" the density function computed out of observations by a level *c*, that varies through the algorithm.

More formally, consider a *p*-dimensional space, $X\subseteq {\mathbb{R}}^{p}$. Let *x*_1_, ..., *x_n _*be a vector of *p*-dimensional observations, $x_{i}\in X$, for *i *= 1, ..., *n*. Starting from this vector, using a method of nonparametric density estimation, we can obtain $\hat{f}(x),x\in X$, i.e. the empirical version of the density *f*(*x*).

There is not a specific method for the nonparametric density estimation related to *pdfCluster*, since the only restriction is that $\hat{f}(x_{i})<\;+\infty$ for all *i *= 1, ..., *n*. This restriction is not limiting, because almost all estimation techniques satisfy it. Following [11], we choose a kernel method with Gaussian kernel and constant smoothing parameter *h *= (*h*_1_, ..., *h_p_*)^⊤^, with $h_{j}={\left(\frac{4}{(p+2)n}\right)}^{1/(p+4)}s_{j},j=1,...,p$, where *s_j _*is the estimated standard deviation of the *j*-th variable. This choice is related to the minimization of the asymptotic integrated mean square error [11]. As suggested by Azzalini and Torelli [11] we slightly shrink *h *toward zero, using a shrinkage factor of 3/4.

Cutting the computed $\hat{f}(x)$ at a level $c\in [0,\max\hat{f}]$, they obtain *m *subspaces ℳ*_k_*, *k *= 1, ..., *m*, of the sample space X. Dropping the observations not belonging to $\cup_{k=1}^{m}{ℳ}_{k}$, they select only those observations *x_i _*such that $\hat{f}(x_{i})>c$. The observations belonging to the same ℳ*_k _*are connected by the Delaunay triangulation (see, e.g., [32]) to form the "cluster cores". Finally, the unallocated observations are allocated by a classification method, based on nonparametric density estimation too: if *x*_0 _is the unallocated observation, the estimated density ${\hat{f}}_{k}(x_{0})$ based on the data already assigned to group *k *is computed, and *x*_0 _is assigned to the group with highest ratio ${\hat{f}}_{k}(x_{0})/\max_{l\neq k}{\hat{f}}_{l}(x_{0})$ Finally, it is important to notice that *pdfCluster *selects by itself the number of clusters.

## Authors' contributions

RDB and DR contributed equally to the paper. Both authors read and approved the final manuscript.
